# Successful Treatment of Relapsing Chronic Eosinophilic Pneumonia With Mepolizumab: A Case Report

**DOI:** 10.7759/cureus.65097

**Published:** 2024-07-22

**Authors:** Lauren Worth, Ali Khreisat, Angela Iacobelli

**Affiliations:** 1 Internal Medicine, Corewell Health William Beaumont University Hospital, Royal Oak, USA; 2 Allergy and Immunology, Corewell Health Beaumont Troy Hospital, Troy, USA

**Keywords:** interleukin-5 inhibitor, chronic dyspnea on exertion, idiopathic chronic eosinophilic pneumonia, chronic eosinophilic leukemia, mepolizumab

## Abstract

We present a case of a 73-year-old male with a five-month history of progressive dyspnea on exertion, cough, and worsening hypoxemia. Initial lab work did not identify peripheral eosinophilia. Chest computed tomography identified extensive ground-glass opacities in the mid-basilar. Diagnostic bronchoscopy showed an eosinophilic-rich bronchoalveolar lavage representing 63% of the total white blood cell count, confirming the diagnosis of chronic eosinophilic pneumonia. No etiology was identified despite extensive diagnostic workup. Our patient had a prolonged course of prednisone taper treatment complicated by frequent hospitalizations, osteopenia, and insomnia. Additionally, his chronic eosinophilic pneumonia relapsed shortly after stopping steroids. In our patient, off-label treatment with mepolizumab, an interleukin-5-inhibiting monoclonal antibody, was associated with symptomatic relief, imaging findings resolution, and remission maintenance without systemic steroids.

## Introduction

Chronic eosinophilic pneumonia (CEP) is an idiopathic disorder that is part of a spectrum of eosinophilic lung diseases, characterized by extensive eosinophilic infiltration of the alveolar lumen, wall, and interstitium, leading to an organized pneumonia that preserves the lung architecture [1]. CEP has an insidious onset manifesting itself with nonspecific constitutional symptoms, cough, dyspnea during exertion, and mild hypoxia that does not respond to supportive treatment or antibiotics [2]. Unlike acute eosinophilic pneumonia, life-threatening respiratory failure from CEP is rare [3]. Systemic steroids are the cornerstone in the treatment of CEP, with a robust resolution of symptoms noted after initiation. However, as in our patient, relapse is often observed after discontinuation of steroids, leading to a prolonged treatment course and increasing the risk of steroids-related complications [4]. Mepolizumab is a humanized monoclonal antibody that targets and inhibits interleukin-5 (IL-5), a vital cytokine for the development and maturation of eosinophils [5]. The off-label use of mepolizumab in the treatment of CEP has been scarcely reported in the literature; however, most cases were not completely off systemic steroids [6]. We present a case of relapsing CEP that has been successfully managed with monthly mepolizumab injections alone.

## Case presentation

**Figure 1:**
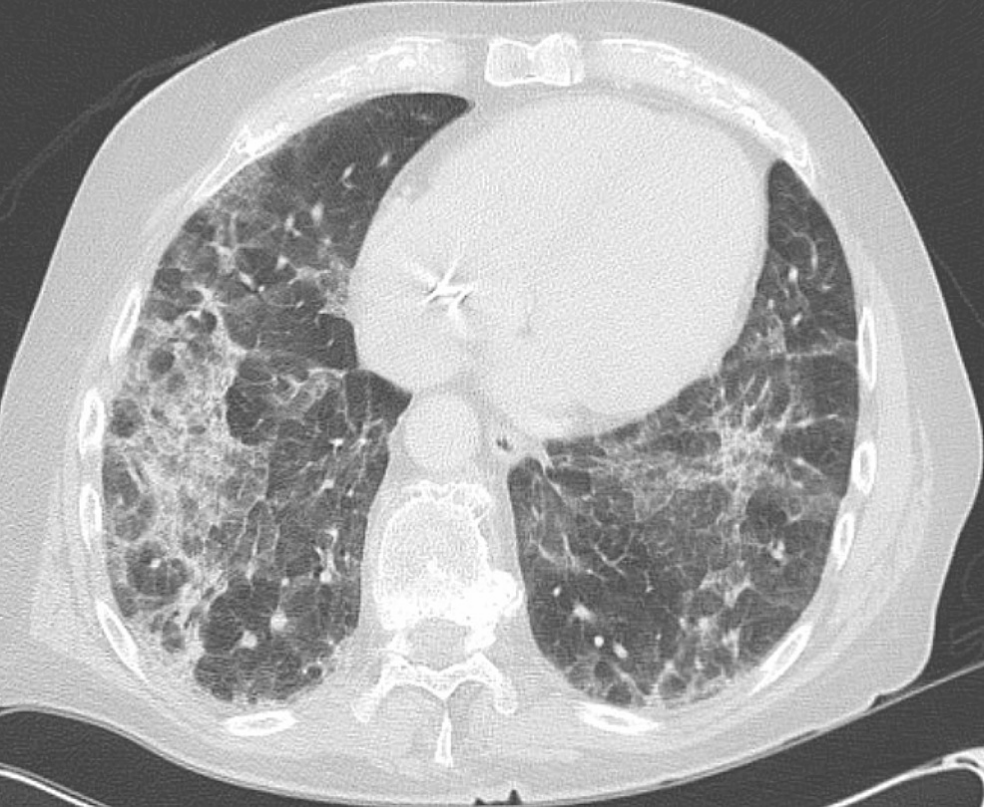
The patient's CT chest with IV contrast showing extensive ground-glass opacities throughout mid-lower lung zones.

A 73-year-old Caucasian man with a medical history of paroxysmal atrial fibrillation, sick sinus syndrome with permanent pacemaker placement, thalassemia trait, and seasonal allergies initially presented to the emergency center for complaints of shortness of breath and dry cough. He reported a respiratory illness five months prior that he had not undergone testing or sought medical care. The patient’s symptoms persisted with worsening shortness of breath on exertion, continued dry cough, and was noted to be hypoxic to 85-88% on his home pulse oximeter. He denied weight loss, fevers, chills, or chest pain but did note some intermittent night sweats. Initial laboratory results were significant for chronic macrocytic anemia, but there was no leukocytosis or eosinophilia. BNP (B-type natriuretic peptide), troponin, procalcitonin, and a comprehensive metabolic panel were all within normal limits. A chest x-ray showed diffuse interstitial opacities. He was hospitalized with pulmonology and cardiology consults and started on antibiotics. A chest computed tomography (CT) with intravenous contrast showed multifocal ground glass opacities throughout the mid to lower lung fields (Figure 1).

A transthoracic echocardiogram showed an ejection fraction of 40% with normal right ventricular function. An infectious workup was negative, including respiratory virus panel, Histoplasma Ag, Blastomyces Ag, Coccidioides Ag, fungal antibody panel, hypersensitivity pneumonitis panels, legionella, *Streptococcus pneumoniae*, and QuantiFERON-TB. Autoimmune workup including Antinuclear antibodies, rheumatoid factor, cyclic citrullinated peptide antibodies, extractable nuclear antibodies, anti-neutrophil cytoplasmic antibodies, and angiotensin-converting enzymes were all within normal limits. Of note, the patient did have an elevated C-reactive protein (CRP) at 18.8 and an erythrocyte sedimentation rate (ESR) of 46. He also underwent serum protein electrophoresis, which showed an increased polyclonal gamma globulin consistent with chronic inflammation. He was started on intravenous methylprednisolone 40 mg daily and his antibiotics were discontinued, and he was discharged home on 2 liters/min nasal cannula with plans for outpatient bronchoscopy.

The results of the outpatient bronchoscopy results revealed a bronchiolar lavage cell count with <2000 RBCs and 375 WBCs with a differential of 63% eosinophils. Bacterial and fungal cultures of the bronchiolar lavage fluid were negative. A transbronchial biopsy showed signs of organizing pneumonia but no granulomas. Carinal lymph node sampling was negative for malignancy or granulomas. He was diagnosed with CEP and started high-dose prednisone 60 mg/day with a plan for prolonged taper. Amiodarone was discontinued as a concern for possible cause. He underwent a high-resolution CT chest two months later, which showed extensive ground glass and nodular infiltrates in both lungs with some progression in the upper lobes and mild mediastinal lymphadenopathy. He was also seen by cardiology regarding his low ejection fraction. A coronary computed tomography angiography (CTA) showed non-obstructive coronary arteries and a cardiac PET scan showed nonspecific increased activity in the mediastinal lymph nodes and in the cardiac walls with a reduced ejection fraction of 34%. He underwent complete pulmonary function tests, which revealed a moderate restrictive defect with total leukocyte count (TLC) 58% of predicted, forced vital capacity (FVC) 70% with no response to bronchodilator, and a severe decrease in diffusion capacity of 24% of predicted. He subsequently was hospitalized for parainfluenza virus and discharged with a prednisone taper, no longer requiring oxygen. Further workup showed an elevated total IgE of 1069 ng/ml and positivity to several Midwest grasses >5, *Penicillium notatum* 1.75 ng/ml, dust mites 0.38 ng/ml, and *Aspergillus fumigatus* 0.47 ng/ml. 

He was then referred to Allergy and Immunology due to his eosinophilic pneumonia, allergies, and elevated IgE. He underwent percutaneous and intradermal allergy testing, which was positive for grasses, weeds, *Fusarium*, dust mites, ragweed, and *Penicillium notatum* but negative for *Aspergillus fumigatus*. Lab work was repeated off prednisone with a CBC w/differential, showing eosinophilia of 4.1 bil/L as well as mild thrombocytopenia of 127/µL and continued chronic macrocytic anemia. A peripheral blood smear showed macrocytic anemia, thrombocytopenia, and eosinophilia without circulating blasts. Other labs were significant for elevated IgE level 3,879, serum tryptase 28, ESR 47, and CRP 14.8. Stool ova and parasite screen, Strongyloides antibody, and Schistosoma antibody were all negative. He was then referred to hematology and underwent a bone marrow biopsy, which was essentially normal and negative for lymphoproliferative disorder, mast cell neoplasm, or other malignancy with a differential of 4% eosinophils. Cytogenetics showed no evidence of a 4q12 FIP1L1::CHIC2::PDGFRa gene rearrangement.

**Figure 2:**
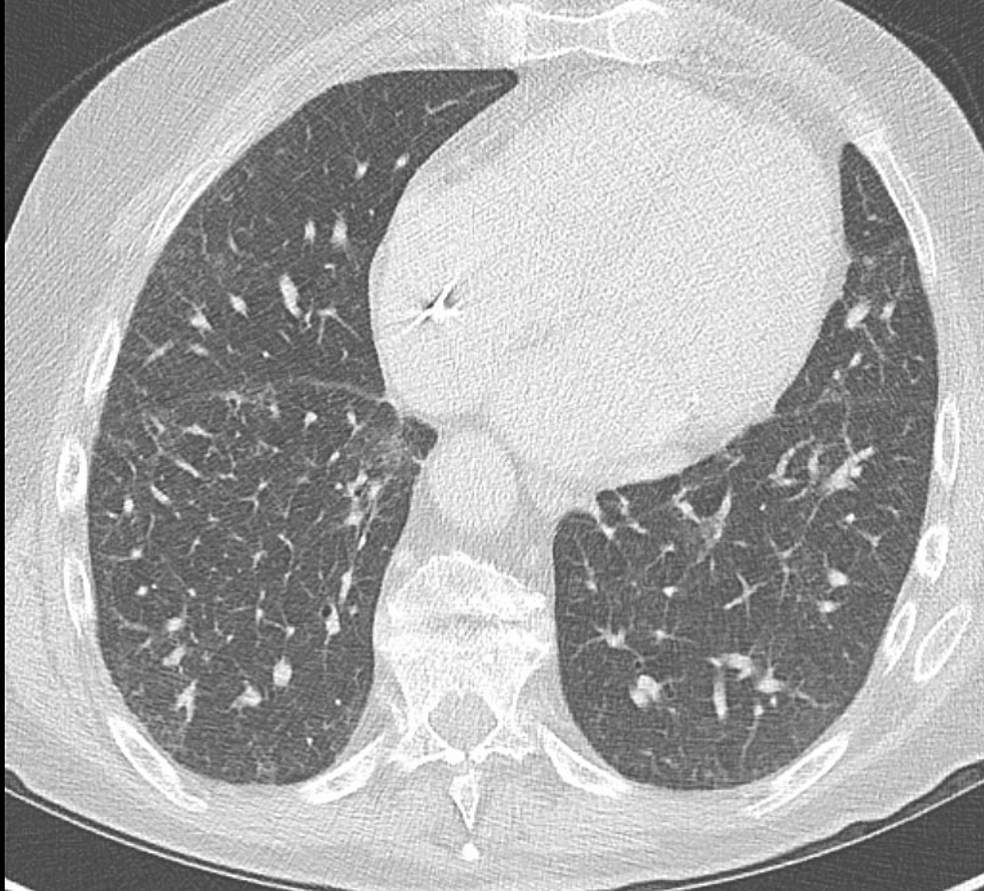
The patient's high-resolution chest CT three months after initiation of mepolizumab showing significant interval improvement in bilateral ground-glass opacities in the mid-lower lung zones.

Upon stopping prednisone, the patient began to experience worsening shortness of breath with exertion, dry cough, night sweats, and itchiness without skin rash. He also noted hypoxia with exertion to 86% and was restarted on home oxygen; CT chest showed recurrence of the basilar ground-glass opacities concerning for CEP relapse. The patient was reluctant to restart oral steroids as he had begun to show signs of osteopenia, insulin resistance, and insomnia. It was decided to trial the patient on mepolizumab at 300 mg monthly as well as restart low-dose prednisone at 5 mg daily. He was clinically improved and no longer required home oxygen a few weeks after starting this regimen. Follow-up chest CT five months after starting mepolizumab showed improvement in interstitial and ground-glass opacities in both lungs (Figure 2). Follow-up lab work showed eosinophilia 0.1 bil/L, IgE level 1,078, tryptase 26, ESR 22, and CRP 0.6. He successfully weaned off steroids and has continued on mepolizumab 300 mg monthly dosing with maintained clinical improvement in symptoms over the course of three months and had sustained remission off systemic corticosteroids over the course of two years. 

## Discussion

In most cases, CEP is idiopathic. Idiopathic CEP (ICEP) is epidemiologically more common in middle-aged women; approximately half of patients with ICEP have a history of atopic diseases such as asthma, allergic rhinitis, or eczema [4]. The first step in the diagnostic workup is to rule out other etiologies, such as allergic bronchopulmonary aspergillosis (ABPA), parasitic infections, drugs, malignancy, and radiation exposure [7]. High-resolution CT imaging usually shows alveolar consolidation patterns and/or ground glass opacities predominantly in peripheral lung zones. The presence of > 25% eosinophils in bronchoalveolar lavage establishes the diagnosis of eosinophilic pneumonia, reducing the need for lung biopsy in some cases [8].

Eosinophils play a pivotal role in inflammation, tissue destruction, and eventually lung fibrosis in patients with CEP. Eosinophil activation in the alveoli and interstitium is followed by the release of cytotoxins such as major basic protein (MBP), eosinophil peroxidase, and transforming growth factor beta (TGF-B), leading to severe tissue damage [9,10]. IL-5 plays a critical role in the hemostasis of eosinophils; activating the IL-5 receptor on the surface of eosinophils helps in the survival and proliferation of eosinophils and migration to the lung tissue [11]. Studies have shown increased levels of IL-5 in the bronchoalveolar lavage of patients with CEP, suggesting a pivotal role of IL-5 in the pathophysiology of this disorder [12]. Corticosteroids are the gold standard therapy for CEP due to their remarkable effect on reducing bone marrow eosinophil production, blood eosinophil count, and tissue inflammation [13]. A dramatic subjective improvement in respiratory symptoms is usually observed after 48 hours of corticosteroid initiation, along with a radiological improvement within one week. However, many patients relapse after discontinuing corticosteroids, leading to a protracted treatment course and corticosteroid-related complications, such as insulin resistance, infections, osteoporosis, and psychiatric disorders [4].

Il-5-inhibiting monoclonal antibodies, such as mepolizumab, have been approved by the FDA for eosinophilic asthma, as an add-on treatment for severe asthma, and for adult patients with eosinophilic granulomatosis with polyangiitis [14]. The off-label use of mepolizumab for CEP has been reported in a few isolated case reports, with less than 100 cases reported in the literature [4,6,15-19]. In those cases, mepolizumab monthly subcutaneous injections with a dose ranging between 100-300 mg were associated with improvement in respiratory symptoms, imaging findings, and reduction in blood eosinophil count within a period of four to six months. However, in many patients in these case reports, corticosteroids were not completely stopped because of fear of relapse. Mepolizumab was also better tolerated than systemic corticosteroids. Our patient showed sustained remission with monthly mepolizumab injections without the need for maintenance systemic corticosteroids over two years.

## Conclusions

Chronic eosinophilic pneumonia (CEP) is a challenging clinical diagnosis in terms of diagnosis, management, and maintenance of remission. Corticosteroids are the cornerstone of inducing and maintaining clinical remission in most cases, leading to increased comorbidities related to long-term exposure to steroids. Randomized controlled trials should be encouraged to investigate the efficacy, tolerance, and sustainability of interleukin-5 monoclonal antibodies compared to systemic corticosteroids in the treatment of CEP, as they could offer a new treatment opportunity for patients with CEP who are corticosteroid-dependent or intolerant.
